# Transthyretin participates in beta-amyloid transport from the brain to the liver- involvement of the low-density lipoprotein receptor-related protein 1?

**DOI:** 10.1038/srep20164

**Published:** 2016-02-03

**Authors:** Mobina Alemi, Cristiana Gaiteiro, Carlos Alexandre Ribeiro, Luís Miguel Santos, João Rodrigues Gomes, Sandra Marisa Oliveira, Pierre-Olivier Couraud, Babette Weksler, Ignacio Romero, Maria João Saraiva, Isabel Cardoso

**Affiliations:** 1 IBMC—Instituto de Biologia Molecular e Celular; 2 i3S—Instituto de Investigação e Inovação em Saúde, Universidade do Porto, Portugal; 3 Faculdade de Medicina, Universidade do Porto, Porto, Portugal; 4 Institut Cochin, INSERM U1016, CNRS UMR 8104, Université Paris Descartes, Sorbonne Paris Cité, Paris, France; 5 Weill Cornell Medical College, Division of Hematology-Medical Oncology, 1300 York Avenue Rm C 606, New York, NY 10065; 6 Department of Life, Health and Chemical Sciences, Open University, Milton Keynes, U.K

## Abstract

Transthyretin (TTR) binds Aβ peptide, preventing its deposition and toxicity. TTR is decreased in Alzheimer’s disease (AD) patients. Additionally, AD transgenic mice with only one copy of the TTR gene show increased brain and plasma Aβ levels when compared to AD mice with both copies of the gene, suggesting TTR involvement in brain Aβ efflux and/or peripheral clearance. Here we showed that TTR promotes Aβ internalization and efflux in a human cerebral microvascular endothelial cell line, hCMEC/D3. TTR also stimulated brain-to-blood but not blood-to-brain Aβ permeability in hCMEC/D3, suggesting that TTR interacts directly with Aβ at the blood-brain-barrier. We also observed that TTR crosses the monolayer of cells only in the brain-to-blood direction, as confirmed by *in vivo* studies, suggesting that TTR can transport Aβ from, but not into the brain. Furthermore, TTR increased Aβ internalization by SAHep cells and by primary hepatocytes from TTR+/+ mice when compared to TTR−/− animals. We propose that TTR-mediated Aβ clearance is through LRP1, as lower receptor expression was found in brains and livers of TTR−/− mice and in cells incubated without TTR. Our results suggest that TTR acts as a carrier of Aβ at the blood-brain-barrier and liver, using LRP1.

Alzheimer’s disease (AD), described for the first time by Alois Alzheimer in 1906, is characterized by progressive loss of cognitive functions ultimately leading to death^1^. Pathologically, the disease is characterized by the presence of extraneuronal amyloid plaques consisting of aggregates of amyloid-beta (Aβ) peptide, and neurofibrillary tangles (NFTs) which are intracellular aggregates of abnormally hyperphosphorylated tau protein^2^. Aβ peptide is generated upon sequential cleavage of the amyloid precursor protein (APP), by beta- and gamma-secretases, and it is believed that an imbalance between Aβ production and clearance results in its accumulation in the brain.

Clearance of Aβ from the brain occurs via active transport at the blood-brain-barrier (BBB) and blood cerebrospinal fluid (CSF) barrier (BCSFB), in addition to the peptidolytic removal of the peptide by several enzymes. The receptors for Aβ at the BBB bind Aβ directly, or bind to one of its carrier proteins, and transport it across the endothelial cell. The low-density lipoprotein receptor-related protein 1 (LRP1) and the receptor for advanced glycation end products (RAGE) are involved in receptor-mediated flux of Aβ across the BBB^3^. Both LRP1 and RAGE are multi-ligand cell surface receptors that, in addition to Aβ, mediate the clearance of a large number of proteins. While LRP1 appears to mediate the efflux of Aβ from the brain to the periphery, RAGE has been strongly implicated in Aβ influx back into the central nervous system (CNS). With increasing age, the expression of the Aβ efflux transporters is decreased and the Aβ influx transporter expression is increased at the BBB, adding to the amyloid burden in the brain.

Transthyretin (TTR), a 55 kDa homotetrameric protein involved in the transport of thyroid hormones and retinol, has been proposed as a protective protein in AD in the mid-nineties, when Schwarzman and colleagues described this protein as the major Aβ binding protein in CSF. These authors described that TTR was able to inhibit Aβ aggregation and toxicity, suggesting that when TTR fails to sequester Aβ, amyloid formation occurs^4^,^5^. Data showing that TTR is decreased in both CSF^6^ and plasma^7^,^8^ of AD patients, strengthen the idea of neuroprotection by TTR. Evidence coming from *in vivo* studies in AD transgenic mice established in different TTR genetic backgrounds^9^,^10^ also suggests that TTR prevents Aβ deposition and protects against neurodegeneration, although the exact mechanism is still unknown. Ribeiro and colleagues reported increased Aβ levels in both brain and plasma of AD mice with only one copy of the TTR gene, when compared to animals with two copies of the gene^11^, suggesting a role for TTR in Aβ clearance. Growing evidence also suggests a wider role for TTR in CNS neuroprotection, including in ischemia^12^, regeneration^13^ and memory^14^.

The presence of TTR in brain areas other than its site of synthesis and secretion – the choroid plexus (CP) and CSF, respectively–in situations of injury, such as ischemia, has been shown using a mouse model with compromised heat-shock response^12^. Authors showed that TTR was not being locally synthesized, but instead should derive from CSF TTR. However, other studies demonstrated TTR synthesis by cortical^15^ or hippocampal neurons both *in vitro*^16^, and *in vivo*^17^, and some hints on its regulation have already been advanced. Kerridge and colleagues showed that TTR is expressed in SH-SY5Y neuroblastoma cell line, and that it is up-regulated by the AICD fragment of amyloid precursor protein (APP), specifically derived from the APP695 isoform. Induced accumulation of functional AICD resulted in TTR up-regulation and Aβ decreased levels^16^. Wang and colleagues reported that TTR expression in SH-SY5Y cells, primary hippocampal neurons and hippocampus of APP23 mice is significantly enhanced by heat shock factor 1 (HSF1)^17^. In any case, TTR is available in the brain and might participate in brain Aβ efflux by promoting BBB permeability to the peptide. With regard to Aβ peripheral elimination, it is known that Aβ bound to ApoE/cholesterol can be incorporated in HDL to be further delivered at the liver for degradation^18^ and curiously, a fraction of TTR is transported in HDL^19^. Furthermore, the liver is the major site for TTR degradation and although its hepatic receptor has never been unequivocally identified, it has been reported that it is a RAP-sensitive receptor^20^. Thus, in this work we assessed the role of TTR in Aβ transport, both from the brain and to the liver.

## Materials and Methods

### Preparation of Aβ1-42 peptides

Aβ1-42 peptide was purchased from Genscript®, dissolved in hexafluoro-2-propanol (HFIP) and kept at room temperature (RT) overnight. The HFIP was removed under a stream of nitrogen and the residue was then dissolved in DMSO at 2 mM. Aβ1-42 labeled with 5-(and-6)-Carboxyfluorescenin (FAM) – FAM-Aβ1-42 – was purchased from AnaSpec and dissolved in Phosphate Buffered Saline (PBS) with 1% NH_4_OH. For *in vitro* experiments, FAM-Aβ1-42 was diluted in either DMEM (Lonza) or WE media (Alfagene®).

### Recombinant TTR production and purification

Human recombinant TTR (h rTTR) was produced in a bacterial expression system using *Escherichia coli* BL21^21^ and purified as previously described^22^. Briefly, after growing the bacteria, the protein was isolated and purified by preparative gel electrophoresis after ion exchange chromatography. Protein concentration was determined by the Bradford Method (Bio-Rad), using bovine serum albumin (BSA) as standard.

TTR was labelled with Alexa 488, using the Alexa Fluor® 488 Protein Labeling Kit (Invitrogen™), following the manufacturers’ instructions.

### TTR depletion from human sera

Human sera from donors, who were informed of the purpose of the study and gave their written consent, were collected in accordance with the approved guidelines. Samples were subjected to affinity chromatography to remove TTR (hTTR), as follows: protein G sepharose beads (GE healthcare) were coupled to the polyclonal rabbit anti-human TTR (Dako) (2 mg of antibody per mL of beads) for 3 hours with shaking. After incubation, beads were washed and incubated 1 hour with fresh crosslinking solution (20 mM dimethyl pimelimidate (DMP) in 100 mM sodium borate pH 9.0) on a shaking platform. Then, beads linked to antibody were transferred to a column and further incubated with 1 mL of human serum for 2 hours at RT. After column packing, TTR depleted serum was collected followed by elution of TTR protein with a suitable Gentle Ag/Ab elution buffer (Thermo Scientific). TTR depletion from human serum was confirmed by western blot.

### Animals

The AD mouse model APPswe/PS1A246E/TTR was used for the quantification of Aβ levels in plasma at different ages. The colony was generated by crossing APPswe/PS1A246E transgenic mice purchased from The Jackson Laboratory with TTR-knockout mice (TTR−/−) (SV129 background) as previously described^9^. In this study, we used cohorts of littermates APPswe/PS1A246E/TTR+/+ (carrying 2 copies of the TTR gene) and APPswe/PS1A246E/TTR+/− (carrying 1 copy of the TTR gene) and APPswe/PS1A246E/TTR−/− (without TTR) female mice aged 3 months. In the next sections, the APPswe/PS1A246E/TTR colony will be referred to as AD/TTR, and the different genotypes APPswe/PS1A246E/TTR+/+, APPswe/PS1A246E/TTR+/− and APPswe/PS1A246E/TTR−/− referred to as AD/TTR+/+, AD/TTR+/− and AD/TTR−/−, respectively.

TTR-wild type (TTR+/+), TTR-heterozygous (TTR+/−) and TTR-knockout (TTR−/−) mice in a SV129 background^23^ were obtained from the littermate offspring of heterozygous breeding pairs, and were used at 2–3 months of age.

Animals were housed in a controlled environment (12-h light/dark cycle; temperature, 22±2 °C; humidity, 45–65%), with freely available food and water. All efforts were made to minimize pain and distress. All procedures involving animals were carried out in accordance with National and European Union Guidelines for the care and handling of laboratory animals and were performed in compliance with the institutional guidelines and recommendations of the Federation for Laboratory Animal Science Association (FELASA) and were approved by the National Authority for Animal Health (DGAV; Lisbon, Portugal).

### Blood collection and determination of Aβ1-42 and Aβ1-40 levels

AD/TTR mice were profoundly anesthetized with an anesthetic combination of ketamine (75 mg/Kg) and medetomidine (1 mg/Kg) by intraperitoneal injection (IP). Blood was collected from the inferior vena cava with syringes with EDTA to obtain plasma, followed by centrifugation at 1000 rpm for 15 minutes at RT. Plasma and sera samples were collected and frozen at −80 °C until used.

Plasma Aβ1-42 and Aβ1-40 levels were quantified using Aβ1-42 Human Ultrasensitive ELISA Kit and Aβ1-40 Human ELISA Kit (Invitrogen™), respectively, according to the manufacturer’s instructions.

### Cell lines and primary hepatocytes culture

The immortalized human cerebral microvascular endothelial cell line (the hCMEC/D3 cell line) was initially produced and characterized by Dr. P.O. Couraud and colleagues and further validated as a model of human BBB by a number of labs worldwide^24^,^25^. Cells were used between passages 25 and 35. Cell culture surfaces were coated with rat tail collagen type I solution (150 μg/mL, Corning life sciences). For culturing, cells were grown in EBM-2 medium (Lonza) supplemented with 5% of fetal bovine serum (FBS) (Gibco™), 1% of penicillin-streptomycin (Lonza), 1.4 μM of hydrocortisone (Sigma-Aldrich), 5 μg/mL of Ascorbic acid (Sigma-Aldrich), 1% of chemically defined lipid concentrate (Gibco™), 10 mM of 4-(2-hydroxyethyl)-1-piperazineethanesulfonic acid (HEPES) (Gibco™) and 1 ng/mL of human basic fibroblast growth factor (bFGF) (Sigma-Aldrich). Cells were cultured in an incubator at 37 °C in a humidified atmosphere of 95% and 5% of carbon dioxide (CO_2_). Cell culture medium was changed every 2–3 days. During experiments, cells were maintained in 0% FBS medium.

SAHep cells, a human hepatoma line not expressing TTR^20^, were grown in Dulbecco’s Modified Eagle Medium (DMEM) (Lonza), supplemented with 10% FBS (Gibco™), 100 U/mL penicillin-streptomycin (Gibco™) and 2mM L-Glutamine (Gibco™).

Primary hepatocytes were derived from TTR+/+, TTR+/− and TTR−/− mice. To obtain these cells, two-step collagenase perfusion of liver was performed with minor alterations^26^. Briefly, a cannula was inserted into the portal vein and perfusion medium (HBSS 1x medium containing 0.025 M HEPES and 2 mM EDTA) was allowed to perfuse through the liver. Then, the vena cava was cut immediately. After 10 minutes, perfusion medium was substituted by collagenase solution (Williams E medium (WE, Gibco™) containing 10% FBS, 3 mM CaCl_2_, 0.01 M HEPES and 50 mg/mL Collagenase type V (Sigma-Aldrich) for another 10 minutes. The entire perfused liver was then removed to a Petri dish containing isolation medium (WE medium containing 10% FBS, 2 mM EDTA and 0.01 M HEPES) proceeding to the next steps of filtering and centrifugations of cell suspension, counting live cells and seeding with attachment medium (WE medium containing 10% FBS and 0.01 M HEPES) for 3 hours. Then the medium was changed to stimulation medium (WE medium containing 2x penicillin-streptomycin, 0.01 M HEPES, 0.04% Fungizone, 0.05 mM Dexamethasone, 1 μM Insulin and 0.05 mM 2 MercaptoEthanol). After 24 hours the medium was renewed and after 48 hours the experiments were performed.

### Cellular uptake and efflux studies

For quantification of Aβ1-42 uptake, hCMEC/D3 cells were plated into a 6-well plate coated with collagen type I and grown to confluence. Cells were washed with PBS and incubated for 5, 10, 15 and 30 minutes at 37 °C, with 500 ng/mL of FAM-Aβ1-42 (100 nM) in presence or absence of h rTTR (7.5 μg/mL, 136 nM). After incubation, cells were washed twice with PBS and enzymatically detached using trypsin (Gibco™), and then resuspended cells were centrifuged at 1000 rpm at RT for 5 minutes. The pellet was washed with PBS and cells recentrifuged as described above. Then the pellet was resuspended with PBS, and if necessary fixed with 4% Paraformaldehyde (PFA). Finally, cells were analyzed in FACS Canto II equipment (BD Biosciences) using Blue laser excitation of 488 nm. The flow cytometry data were analyzed using the Flowjo workstation.

For efflux studies, hCMEC/D3 cells were initially incubated with FAM-Aβ1-42 for 10 minutes and then the media was removed and replaced with FAM-Aβ1-42 -free media, and further incubated for 10 and 20 minutes, at 37 °C. Finally, cells were analyzed by flow cytometry, as described above.

For the study with hepatocytes, SAHep cells were grown in 6-well plates coated with collagen type I and after 24 hours cells were incubated with FAM-Aβ1-42 in the presence or absence of h rTTR (136 nM) for 3 hours at 37 °C. For primary hepatocytes, after 48 hours of seeding, cells were incubated with FAM-Aβ1-42 (100 nM), with the advantage of no need for TTR addition. For hepatocytes derived from TTR−/− mice, we also examined the effect of h rTTR on FAM-Aβ1-42 internalization. FAM-Aβ1-42 inside SAHep cells and primary hepatocytes was measured by flow cytometry (FACS Canto II, BD Biosciences).

TTR uptake by hCMEC/D3 cells was studied by incubating cells with Alexa488-TTR for 10, 20 and 30 minutes. Cells were then fixed and observed under the microscope Zeiss Axio Imager Z1 equipped with an Axiocam MR3.0 camera and Axiovision 4.7 software.

### hCMEC/D3 permeability to Aβ peptide and TTR

For BBB transport experiments, hCMEC/D3 cells were seeded on type I collagen pre-coated transwells filters (polyester 12 well, pore size 0.4 μm; Costar). Culture medium was changed every 3 days and assays were performed 10 days after seeding.

To identify filters in which apical medium leaked into the basolateral medium, apical media were supplemented with 0.25 mg/mL Fluorescein isothiocyanate (FITC)-labeled dextran (molecular mass of 70 KDa; Sigma-Aldrich) before transport studies. The concentration of FITC-labeled dextran was determined fluorometrically (excitation: 492 nm; emission: 518 nm) in the collected basolateral media using the same microplate reader as described previously. Wells in which the basolateral FITC-labeled dextran concentration exceeded 125 ng/mL, indicating that the cell layer had been disrupted, were excluded from analysis. The FITC-labeled dextran concentration was calculated from a standard curve ranging from 0.25 mg/mL to 62.5 ng/mL.

Thus, transport studies were performed by supplementing the basolateral compartment (brain side) with 500 pg/mL Aβ1-42 (0.111 nM), whereas h rTTR 7.5 μg/mL (136 nM) was added either to the basolateral or to the apical compartment. Similar experiments were performed using human sera (2.5%) as the source of hTTR and to mimic the absence of this protein we used TTR-depleted sera (3%). Experiments were also performed using cells incubated only with human sera or TTR-depleted sera in apical and basolateral sides, in order to evaluate the passage of TTR across hCMEC/D3 cells in absence of Aβ1-42. Then, filters were incubated at 37 °C, and after 3, 10, 24 and 48 hours samples were drawn from both sides and were replaced with the same amount of 0% FBS medium. At the end of experiment, Aβ1-42 levels in both compartments were quantified using the ELISA kit (Invitrogen™) following manufacture’s recommendations. TTR was also evaluated by ELISA as will be described later.

### TTR clearance *in vivo*

TTR ability to cross the BBB, in both directions, was studied *in vivo* using TTR −/− mice and injecting h rTTR. To assess the brain-to-blood permeability, immediately before the injection, mice were weighed and anesthetized with intraperitoneal injection of an anesthetic combination of ketamine and medetomidine (7.5 mg/Kg and 0.1 mg/Kg, respectively) and placed in a stereotaxic apparatus (Stoelting Co.). The cranium was exposed using an incision in the skin and one small hole was drilled through the cranium over the right lateral ventricle injection site to the following coordinates: mediolateral −1.0 mm, anterior-posterior −0.22 mm and dorsal-ventral −1.88 mm, from bregma. Then, 10 μg of h rTTR were injected into the brain using a 10 μL motorized syringe (Hamilton Co.) connected to a 30 gauge needle (RN Needle 6 pK, Hamilton Co.) at a rate of 0.75 μL/min (4 μL final volume). After injection, the microsyringe was left in place for 3 minutes to minimize any backflow, and then the incision was closed with sutures (Surgicryl), and the wound was cleaned with 70% ethanol. After surgery, the animals were kept warm, using a warming pad, and blood samples were collected by the tail vein after 20, 40 and 60 minutes, in a capillary tube (previously coated with EDTA). At the time of sacrifice (after 60 minutes), the mice were re-anesthetized with 75 mg/Kg ketamine and 1 mg/Kg medetomidine, and after total absence of reflexes in the paw and tail, mice were perfused through the injection of sterile PBS pH 7.4 via the inferior vena cava until the liver becomes blanched. Then, the brain was rapidly collected and frozen at −80 °C until use.

To assess the blood-to-brain permeability, 10 μg of h rTTR were injected in the tail vein, and blood samples were collected after 20, 40 and 60 minutes. At 60 minutes, and after perfusion as described above, CSF and brain were also collected.

To determine TTR levels, brains were weighted and homogenized in 750 μL of 50 mM TBS pH 7.4 containing protease inhibitor cocktail. After centrifugation for 20 minutes at 14000 rpm at 4 °C, supernatants were collected. TTR concentration in brain, CSF and plasmas was determined by ELISA.

### ELISA for human and mouse TTR

Sandwich ELISA was used to determine h rTTR and hTTR concentration in apical and basolateral sides of transwells in BBB transport studies, as well as h rTTR in brain, CSF and plasma from the *in vivo* TTR clearance studies. 96-well-plates (Maxisorp-Nunc) were coated overnight at 4 °C with a polyclonal rabbit anti-human TTR antibody (1:200 dilution) in 50 mM carbonate/bicarbonate buffer. After washing with PBS-T, 96-wells were blocked with a commercial blocking reagent, PowerBlock (Biogenex), for 2 hours at RT, and after this, samples and standards were applied to the wells during 2 hours at RT. Then, 96-Wells were incubated, 1 hour at RT, with a sheep anti-human TTR antibody (1:2500; Abcam) followed by incubation with anti-sheep IgG alkaline phosphatase antibody produced in donkey (3:10000; Sigma-Aldrich) for 1 hour at RT. Development was performed with SigmaFAST^TM^ p-nitrophenyl phosphate (Sigma-Aldrich). The TTR concentration was calculated from a standard curve ranging from 1.79 to 86 ng/mL. The percentage of TTR that moved on to the opposite side to that on which it was initially placed, was calculated.

Mouse TTR (moTTR) in supernatants of primary hepatocytes was quantified using Mouse Prealbumin ELISA Kit (MyBioSource) according to the manufacturer’s instructions. Data were expressed in mg/L.

### Incubation of hCMEC/D3 and hepatoma cells lines with TTR

hCMEC/D3 and SAHep cells were incubated with their respective media in the absence or presence of h rTTR (2 μM), for 1 hour and further analyzed by immunofluorescence. Alternatively, cells were incubated with h rTTR (2 μM) for 24 hours and collected in Trizol (Alfagene®) for further analysis by qRT-PCR.

### Immunofluorescence for claudin-5, occludin and LRP1

hCMEC/D3 were grown to confluence on glass coverslips (Thermo Scientific) coated with rat tail collagen type I solution and then washed in PBS and fixed with methanol 15 minutes at RT (for tight junctions) or in PFA 4% 30 minutes in 4 °C (for LRP1). Fixed cells were permeabilized with 0.1% TritonX-100 and blocked with 5% BSA followed by incubation with primary antibodies against claudin-5 (Rabbit anti Claudin5, Abcam, 1:200), occludin (Rabbit anti Occludin, Invitrogen™, 1:50) or LRP1 (Rabbit anti LRP1 antibody, Abcam 1:100). After washing, cells were incubated with Alexa Fluor-488 donkey anti-rabbit IgG antibody (Life technologies, 1:1000). Then, coverslips were mounted with Fluoroshield™ with DAPI (Sigma-Aldrich) and visualized and photographed with Confocal Microscope (Leica SP2 AOBS SE) for tight junctions or by Zeiss Axio Imager Z1 equipped with an Axiocam MR3.0 camera and Axiovision 4.7 software.

### Protein extraction

Livers were homogenized in lysis buffer (20 mM MOPS pH 7.0; 2 mM EGTA; 5 mM EDTA; 30 mM sodium fluoride; 60 mM β-glycerophosphate pH 7.2; 20 mM sodium pyrophosphate; 1 mM sodium orthovanadate; 1% triton X-100), 1 mM phenylmethylsulphonyl fluoride (PMSF) and protease inhibitors (GE healthcare), followed by 20 minutes on ice. Extracts were then centrifuged at 14000 rpm at 4 °C for 20 minutes, and supernatants were used for protein analysis. To extract protein from brains, tissues were weighted and homogenized in 750 μL of 50 mM TBS pH 7.4, containing protease inhibitor cocktail. After centrifugation for 20 minutes at 14000 rpm at 4 °C, supernatants were collected.

Total protein concentration was quantified in all extracts by the Bradford Method (Bio-Rad), using BSA as standard.

### Western-Blot analysis

The presence of TTR in human serum after depletion, as well as LRP1 total levels in brains and livers was studied by western blot analysis. Protein extract denatured samples (30–50 μg) were separated in SDS-PAGE gels (10% for LRP1 and 15% for TTR). Proteins were then transferred to nitrocellulose membrane (Whatman^TM^ Ge healthcare – Protan BA 83), using a wet system (Bio-rad Criterion Blotter). The membranes were blocked 1 hour at RT with 5% powered skimmed milk in PBS containing 0,05% Tween-20 (PBS-T). After blocking, membranes were then incubated with primary antibodies in 3% powered skimmed milk/PBS-T against the proteins under study: rabbit anti TTR (DAKO, 1:2000), rabbit anti LRP1 (Abcam, 1:15000), mouse anti Tubulin (Sigma-Aldrich, 1:2000), mouse anti β-Actin (Sigma-Aldrich, 1:3000). Then washed membranes were incubated for 1 hour at RT with sheep anti-rabbit (The binding Site; 1:10000) or anti-mouse (The binding Site; 1:2500) immunoglobulins conjugated with horseradish peroxidase in 3% powered skimmed milk/PBS-T. The blots were developed using Clarity^TM^ Western ECL substrate (Bio-rad) and proteins were detected and visualized using a chemiluminescence detection system (ChemiDoc, Bio-rad).

### qRT-PCR for LRP1 levels

Mice with different TTR genetic backgrounds were perfused with PBS and their brains and livers were collected. One hemisphere of the brains and a piece of liver approximately of 60 mg were homogenized and Total RNA isolated using Trizol (Alfagene®). RNA concentrations were measured by a NanoDrop 1000 spectrophotometer and stored at −80 °C until further use. hCMEC/D3 and SAHep cells were also collected in Trizol to be further analyzed. For the reverse transcription to cDNA, 4 μg of RNA was used with the SuperScript First-Strand Synthesis System (Invitrogen™ or Enzytech). The reaction mix was then subjected to quantitative real-time PCR with the SYBR Green reporter (iQ SYBR Green supermix, *BioRad*) to detect levels of LRP1 and Glyceraldehyde 3-phosphate dehydrogenase (GAPDH, as reference gene). The primers used were as follows: LRP-1 sense 5′-CGAGGAGCAGGTTGTTAG-3′; LRP-1 antisense 5′-CAGAAGCAGCAGGAGAAG-3; GAPDH sense 5´-GCCTTCCGTGTTCCTACC-3′, GAPDH antisense 5′-AGAGTGGGAGTTGCTGTTG-3′. Reactions were run in a Bio-Rad iCycler. The relative levels of expression were quantified and analyzed by Bio-Rad iQ5 software. Data were calculated using the ΔCT Method Using GAPDH as a Reference Gene, before statistical analysis was performed.

### Statistical analysis

All quantitative data were expressed as mean±SEM. Initially data was assessed whether it followed a Gaussian distribution. When found to follow a Gaussian distribution, differences among conditions or groups were analyzed by one-way ANOVA with a Sidak’s multiple comparisons test. In the cases of non-Gaussian distribution, differences among conditions were analyzed by non-parametric Kruskal-Wallis test and comparisons between two groups were made by Student t-test with a Mann Whitney test. P-values lower than 0.05 were considered statistically significant. Statistical analyses were carried out using Graphpad Prism 5 software for Windows.

## Results

### Characterization of the hCMEC/D3 cell line

The hCMEC/D3 cell line represents a valid and powerful *in vitro* tool as a BBB model, and presents a less expensive and more logistically feasible alternative to primary hBMEC cells^24^,^25^. Thus, our first step was the validation of the hCMEC/D3 model by characterizing this cell line regarding two critical features for our studies: BBB integrity and LRP1 expression.

**Figure 1:**
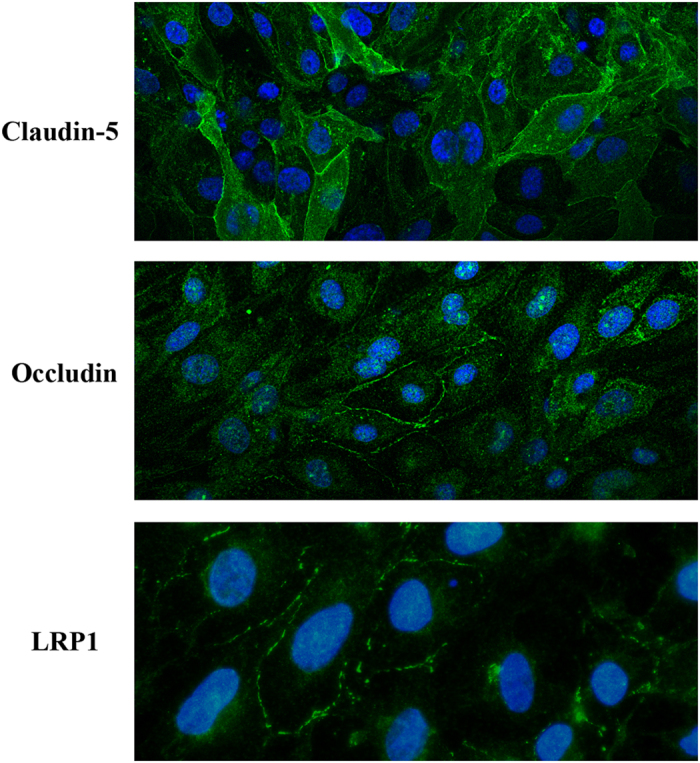
Immunofluorescence localization of TJs components Claudin-5 and Occludin, and of LRP1, in hCMEC/D3.

In the context of endothelial cell tight junctions (TJ), hCMEC/D3 cells were tested for claudin-5 and occludin expression by immunofluorescence. As shown in Fig. 1, hCMEC/D3 cells are positive for TJ structural proteins, claudin-5 and occludin, showing the expected membrane localization (as previously described). These results indicate that the integrity, tightness and structure, as well as the paracellular contact between endothelial cells are guaranteed by these TJ proteins. Along with other TJ proteins expressed by hCMEC/D3, claudin-5 and occludin ensure, with high efficiency, the control of transport across the cells monolayer.

The expression of the efflux transport receptor LRP1 by the hCMEC/D3 cell line is a key factor when validating this model, both for BBB studies purposes and for Aβ transport research. Thus, we performed immunofluorescence analysis to verify if LRP1 exists in the hCMEC/D3 cells. Our results show that LRP1 is expressed in these cells ensuring the Aβ transport through the cells monolayer (Fig. 1).

### Effect of TTR in Aβ1-42 internalization by hCMEC/D3

Aβ1-42 is transported across the BBB, as expected, and is internalized by hCMEC/D3 cells. We firstly investigated FAM-labelled Aβ1-42 (FAM-Aβ1-42, 500 ng/mL)) uptake by these cells in the absence and presence of human recombinant TTR (h rTTR) (7.5 μg/mL), and analysed the results by flow cytometry.

**Figure 2:**
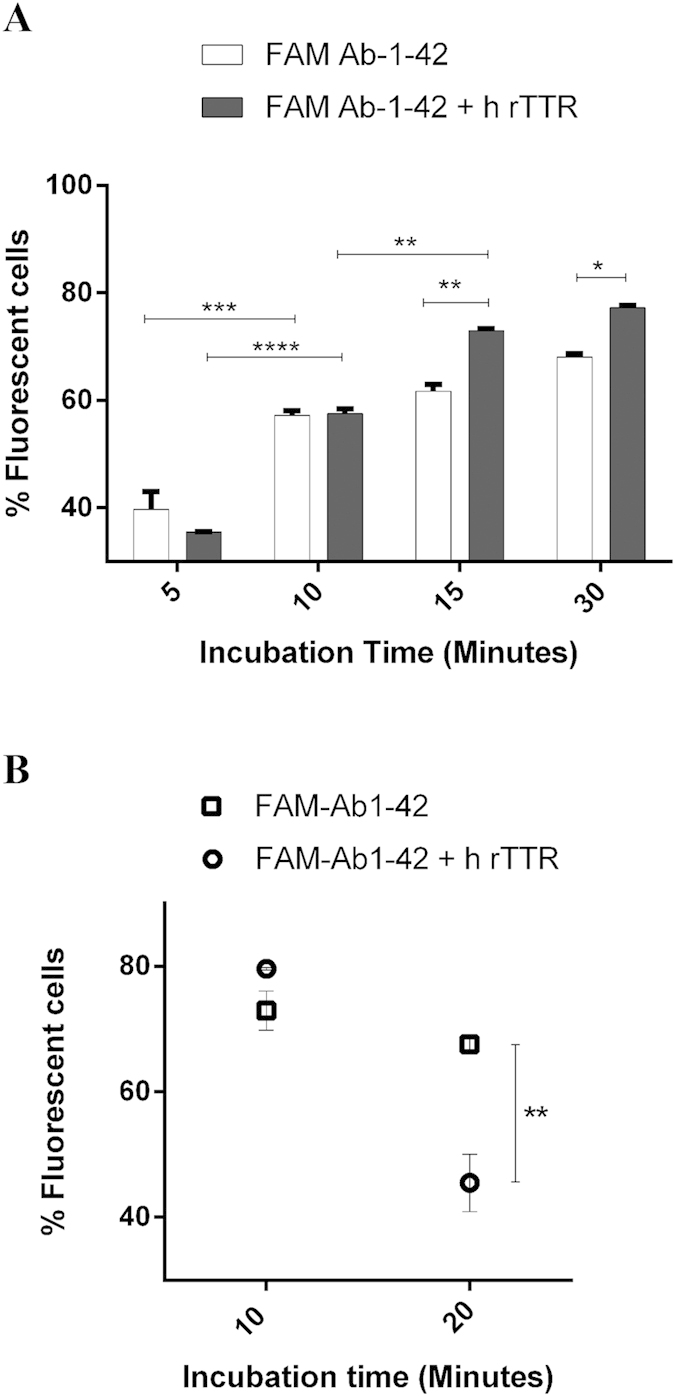
Interaction of FAM-Aβ1-42 with hCMEC/D3 cells in the presence and absence of TTR assessed by flow cytometry: (**A**) Internalization levels of FAM-Aβ1-42 by hCMEC/D3 cells in the presence of h rTTR (white columns) was significantly higher than in the absence of the protein (black columns) after 15 and 30 minutes of incubations. (**B**) Efflux of FAM-Aβ1-42 from hCMEC/D3 measured after 10 minutes of incubation with the peptide was significantly increased at 20 minutes post-replacement with fresh FAM-Aβ1-42-free media, in the presence of h rTTR. N = 3 for each condition and data are expressed as mean±SEM.

Cells were incubated with FAM-Aβ1-42 at 37 °C producing a rapid uptake of the peptide (Fig. 2A). After 5 minutes of incubation, 35–39% of the cells were fluorescent and after an additional 5 minutes (10 minutes incubation) a significant increase was already measured as over 57% of the cells were fluorescent, although differences between the presence and absence of TTR were not significant. However, after 15 minutes the presence of TTR significantly increased Aβ internalization resulting in about 73% fluorescent cells, in contrast to 61.7% incubated in the absence of TTR (Fig. 2A). Finally after 30 minutes of incubation, and although the difference between internalization levels at 15 and 30 minutes was not statistically significant, FAM-Aβ1-42 internalization was significantly higher in the presence of TTR.

Next to investigate the fate of internalized Aβ, we performed an efflux assay. For that, hCMEC/D3 cells were firstly incubated with FAM-Aβ1-42 for 10 minutes, in the absence or presence of h rTTR and then the media were replaced with fresh Aβ-free media. Cells were further incubated at 37 °C and levels of FAM-Aβ1-42 inside cells were measured by flow cytometry, after 10 and 20 minutes. Figure 2B depicts the results showing that in the presence of TTR, FAM-Aβ1-42 effluxes significantly faster than in the absence of this protein, after 20 minutes (45.5% and 67.6% fluorescent cells, respectively).

### Effect of TTR in hCMEC/D3 brain-to-blood permeability to Aβ1-42 peptide

**Figure 3:**
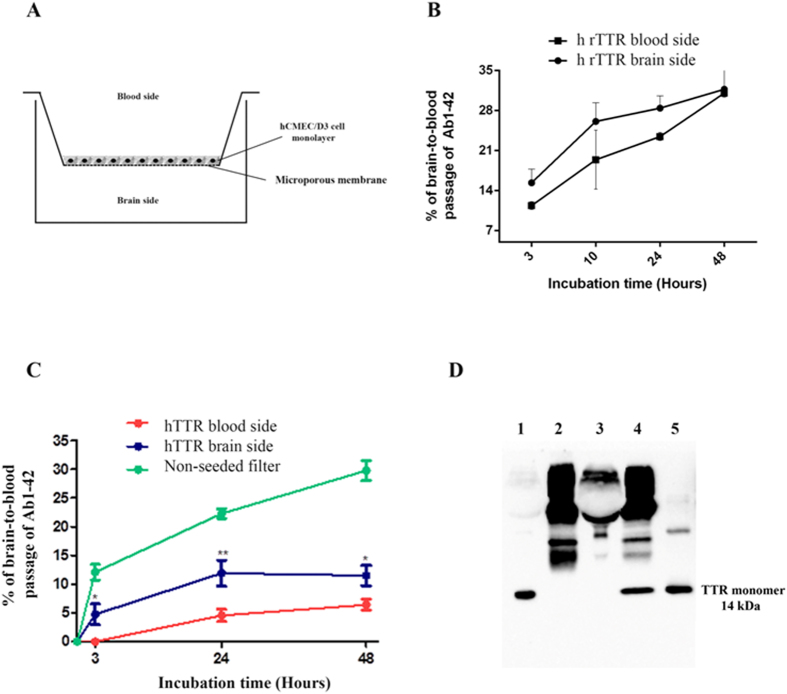
Brain-to-blood permeability of hCMEC/D3 cells to Aβ1-42: (**A**) Schematic representation of the transwell system used showing the brain and blood sides; Aβ1-42 peptide was always added to the brain side, whereas TTR was added either to the brain or to the blood sides. (**B**) Brain-to-blood permeability was increased in the presence of h rTTR although without reaching significant differences. However, in the presence of (**C**) hTTR present in sera, brain-to-blood permeability of hCMEC/D3 cells to Aβ1-42 was significantly increased after 3 hours up to 48 hrs. As a control, Aβ peptide was also added to non-seeded filters to show free passage of the peptide when compared to cell-seeded ones. N = 3 for each condition and data are expressed as mean±SEM. To mimic the absence of TTR, we used TTR-depleted human sera obtained after affinity chromatography, and further analysed by western blot (**D**) lanes 1- human sera; 2- protein G sepharose beads/anti-human prealbumin antibody; 3-human sera TTR-depleted; 4-Eluted TTR; 5-r hTTR.

In order to investigate the effect of TTR in Aβ1-42 transport across a monolayer of cells, acting as a model of the BBB as previously described, Aβ1-42 transport experiments were performed in hCMEC/D3 cultured in transwells inserts, as shown in Fig. 3A. Cells were grown for 10 days until reaching maximal confluence and allowing TJ formation. Thus, at this point, the cell monolayer should show restricted paracellular permeability, and its confirmation was done using FITC-labelled dextran as a low molecular weight paracellular diffusion marker. In this approach, FITC-labelled dextran 0.25 mg/mL was added to the apical chamber, and then incubated for 1 hour. Wells in which FITC-labelled dextran exceeded 125 ng/mL on the basolateral chamber were considered to have the monolayer disrupted and thus were excluded from the experiment.

We added h rTTR either to the brain or to the blood side, whereas Aβ1-42 was always added to the brain side. Results are displayed in Fig. 3B and show increased permeability of the hCMEC/D3 monolayer to Aβ1-42, when h rTTR is in the brain side, as compared to the levels of Aβ1-42 passage when h rTTR is in the blood side, although the differences were not statistically significant.

To further evaluate the effect of TTR in Aβ1-42 transport across the BBB and in order to obtain a more complex environment in hCMEC/D3 model, we performed the same transwell experiments but using human sera as source of hTTR (TTR concentration 7.5 μg/ml). To mimic the absence of TTR, we used human sera after TTR depletion by affinity chromatography (Fig. 3D). Again, hTTR present in the brain side promoted significant Aβ1-42 transport across the hCMEC/D3, as compared to the situation where hTTR was in the blood side (Fig. 3C). This suggests that TTR participates in Aβ1-42 efflux from the brain through a mechanism that implies TTR/Aβ interaction at the BBB or in its vicinity.

### Brain permeability to TTR

**Figure 4:**
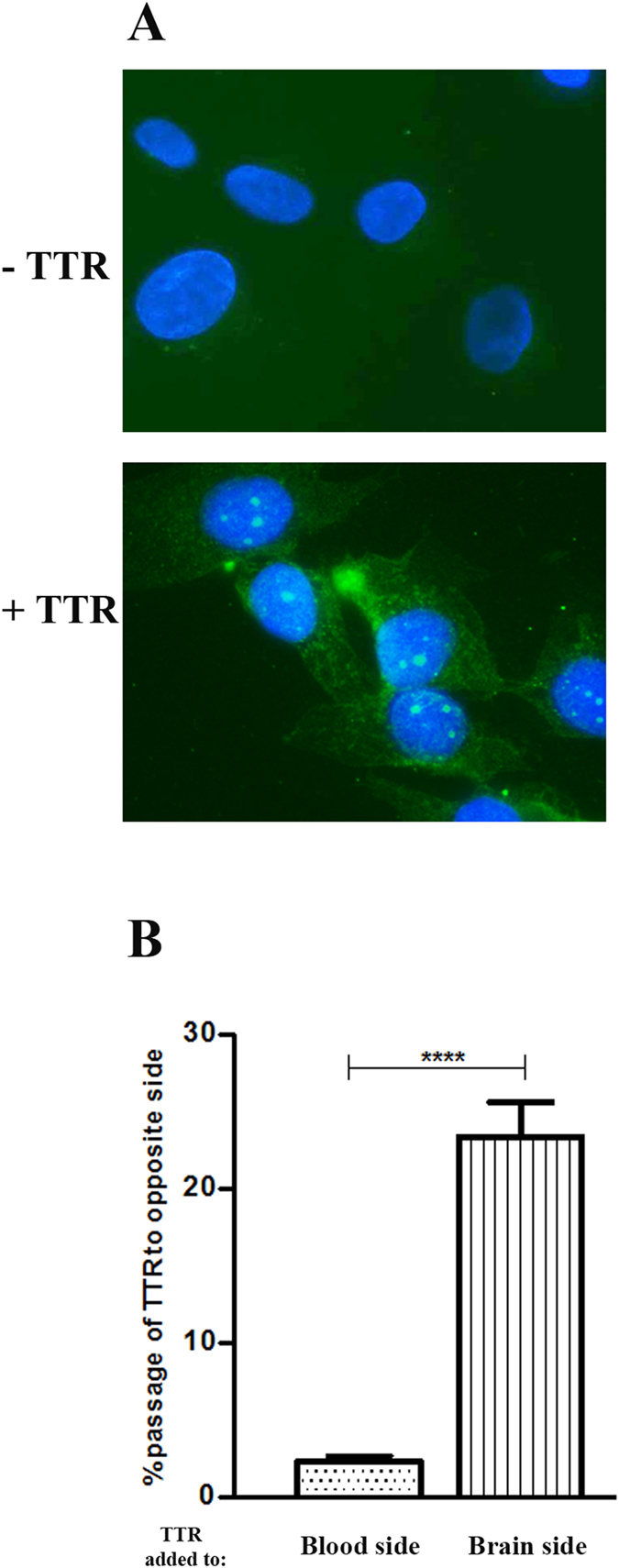
Permeability of hCMEC/D3 cells to TTR: (**A**) hCMEC/D3 cells internalize TTR, as assessed by fluorescence microscopy. (**B**) hCMEC/D3 cells are permeable to TTR in the brain-to-blood direction but not in the blood-to-brain direction. N = 3 for each condition and data are expressed as mean±SEM.

Given our evidence in TTR-assisted Aβ transport and to clarify if TTR might be co-transported during such process, we assessed TTR internalization by hCMEC/D3 cells, and as shown in Fig. 4A, TTR was uptaken by these cells.

We next investigated if TTR could cross the hCMEC/D3 monolayer and to assess this, hTTR was added either to the apical or basolateral compartment of the transwells. TTR was then quantified in the media of both chambers and analysed as % TTR that passed to the opposite side. As shown in Fig. 4B, TTR crosses the monolayer in the brain-to-blood direction but not in the blood-to brain direction. This suggests TTR is using a receptor with main expression in the basolateral membrane of the hCMEC/D3 cells.

To confirm these results, we also evaluated TTR clearance *in vivo*, using TTR−/− mice injected with h rTTR, either intracranially (IC) in the right lateral ventricle or intravenously (IV) in the tail vein. As displayed in Table 1, TTR injected in the brain rapidly reached the periphery as TTR was easily detected in blood, whereas mice injected IV showed negligible levels of the protein in the CSF and brain. Thus, this data corroborates the results obtained in the transwell experiments. This also suggests that TTR can favour Aβ brain efflux but cannot favour its influx, contributing to neuroprotection in AD.

### Effect of TTR in Aβ1-42 and Aβ1-40 in AD transgenic mice

Previous work using an AD transgenic model (APPswe/PS1A246E) with different TTR genetic backgrounds (AD/TTR) has demonstrated that Aβ1-42 plasma levels are increased in 7-month old TTR+/− female mice, when compared to TTR+/+ animals^11^, suggesting a role for TTR in Aβ peripheral clearance.

**Figure 5:**
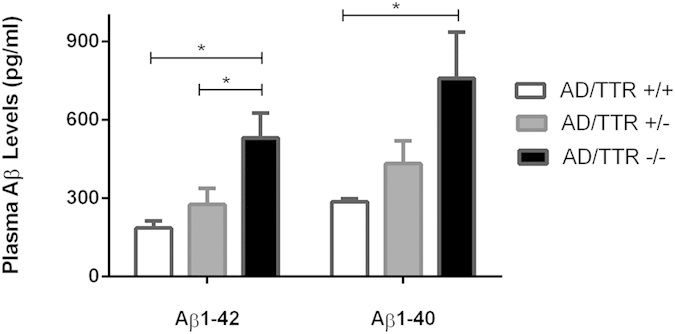
Effect of TTR genetic reduction in plasma Aβ1-42 and Aβ1-40 levels: Results are shown for 3-month old female mice with three distinct genotypes for TTR: AD/TTR+/+ (N = 5 for Aβ1-42; N = 4 for Aβ1-40), AD/TTR+/− (N = 6 for Aβ1-42; N = 4 for Aβ1-40) and AD/TTR−/− (N = 5 for Aβ1-42; N = 4 for Aβ1-40).

In this work, to obtain a better knowledge on the effect of TTR in plasma Aβ peptide levels, we extended the study by evaluating not only Aβ1-42 but also Aβ1-40 levels in 3-months old AD/TTR+/+, AD/TTR+/− and AD/TTR−/− female mice. Results are depicted in Fig. 5 and show a negative correlation between TTR and both Aβ1-42 and Aβ1-40. Differences between AD/TTR+/+ and AD/TTR−/− mice were found to be statistical significant for both Aβ peptides. In addition, for Aβ1-42 statistical significant differences were also observed between AD/TTR+/− and AD/TTR−/−.

Taken together, our results suggest that TTR influences plasma Aβ by reducing its levels.

### Effect of TTR in Aβ1-42 internalization by SAHep cells and primary hepatocytes

**Figure 6:**
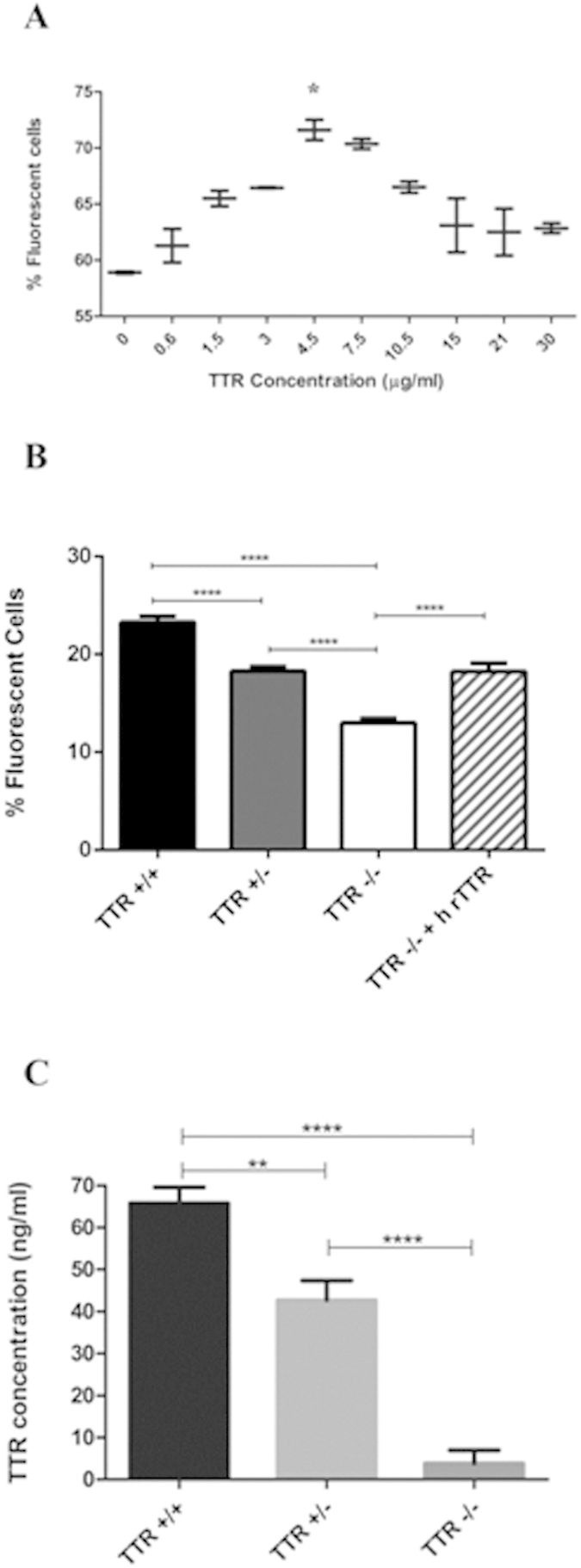
Effect of TTR in Aβ peptide internalization by hepatocytes: (**A**) FAM-Aβ1-42 internalization by SAHep cells, in the absence or presence of increasing concentrations of h rTTR, as measured by flow cytometry. TTR concentrations up to 4.5–7.5 μg/mL resulted in increased Aβ internalization by cells. N = 3 for each condition. (**B**) Flow cytometry of primary cultures of hepatocytes derived from mice with different genetic TTR backgrounds; hepatocytes derived from TTR+/+ mice showed significantly more internalization of FAM-Aβ1-42 than those derived from TTR+/− and from TTR−/−. N =  11, N = 8, N = 14, N = 6 for hepatocytes derived from TTR +/+, TTR +/−, TTR −/− and h rTTR treated TTR −/− mice, respectively. (**C**) moTTR levels in supernatants of primary hepatocytes measured by ELISA confirmed the genetic reduction in TTR+/− which showed about half of the TTR in TTR+/+, while TTR−/− produced no TTR protein. N = 7 for TTR+/+ and −/− mice and N = 5 for TTR +/−.

Aβ is known to also be delivered at the liver for degradation; therefore, we analysed the effect of TTR in FAM-Aβ1-42 internalization using the SAHep cell line. Uptake of Aβ1-42 peptide increased in the presence of h rTTR showing a positive correlation between Aβ uptake and h rTTR concentration, reaching a maximum of 70% when using 4.5–7.5 μg/mL of TTR in 3 hours (Fig. 6A).

To further study the effect of TTR in Aβ1-42 uptake by hepatocytes, and in order to avoid addition of exogenous TTR (since hepatocytes produce TTR), we prepared primary cultures of hepatocytes derived from mice with different TTR genetic backgrounds (TTR+/+, TTR+/− and TTR−/−). TTR secretion was evaluated by ELISA revealing values of approximately 70 and 40 ng/mL for TTR+/+ and TTR+/−, respectively, over a period of 3 hours (Fig. 6C). TTR−/− hepatocytes did not produce TTR, as expected.

As for Aβ1-42 uptake, we observed that TTR facilitated peptide internalization by primary hepatocytes as differences were statistically significant between genetic backgrounds (Fig. 6B). Importantly, addition of h rTTR to TTR−/− hepatocytes partially rescued the phenotype as internalization values equalized those of TTR+/− cells.

### Influence of TTR on LRP1 levels

**Figure 7:**
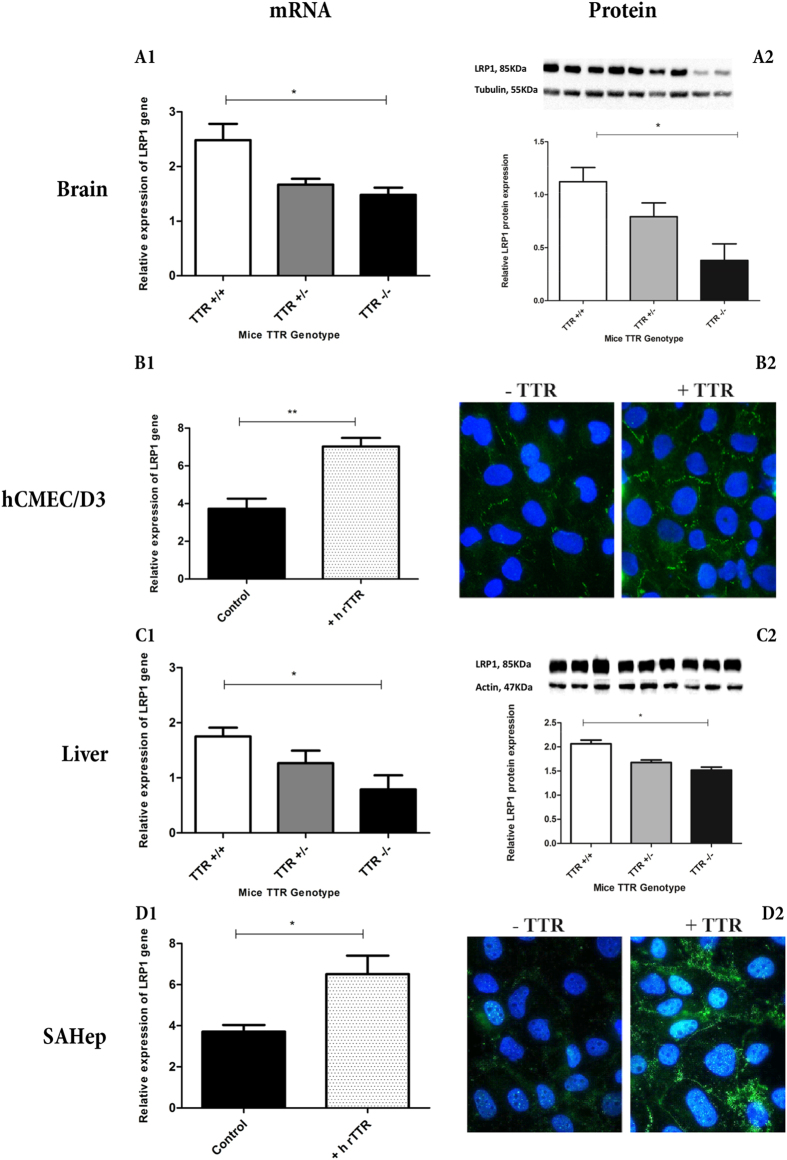
LRP1 expression in the brain, liver and cell lines assessed by qRT-PCR, western blot and immunofluorescence: LRP1 levels investigated in the brains from TTR+/+, TTR+/− and TTR−/− mice by (**A1**) qRT-PCR (n = 4) and (**A2**) by western blot (n = 3), showed to correlate directly with TTR levels. hCMEC/D3 cells (n = 3) incubated with TTR showed higher amounts of (**B1**) mRNA and (**B2**) protein than cells without TTR. Similarly, livers of TTR+/+ mice expressed more LRP1, both (**C1**) mRNA (n = 4) and (**C2**) protein (n = 3), than of TTR−/− mice. (**D1**) qRT-PCR for LRP1 in SAHep cells incubated with exogenous h rTTR increased their LRP1 mRNA levels (n = 3). (**D2**) Upon incubation with TTR, SAHep cells increased their LRP1 protein levels.

We firstly assessed LRP1 expression by qRT-PCR in total brain extracts of TTR+/+, TTR+/− and TTR−/− mice, and observed significant differences in the expression of this receptor: brains from TTR+/+ mice expressed LRP1 in significantly higher levels than brains from TTR−/− animals (Fig. 7A1). These results were corroborated by measuring LRP1 protein levels by western blot (Fig. 7A2).

To further understand the importance of TTR in regulating LRP1 levels in the context of Aβ transport across the BBB, we incubated hCMEC/D3 cells with h rTTR and investigated LRP1 expression by qRT-PCR. As depicted in Fig. 7B1, hCMEC/D3 incubated with TTR displayed higher LRP1 expression, thus confirming the regulation of LRP1 by TTR in these endothelial cells; these results were also corroborated by protein levels, as evaluated by immunocytochemistry (Fig. 7B2)

Similarly to the internalization studies, we also evaluated the ability of TTR to regulate LRP1 levels in hepatocytes by performing qRT-PCR studies in livers from TTR+/+, TTR+/− and TTR−/− mice, as well as in the hepatocyte cell line, SAHep cells. Similarly to the brains, livers from TTR+/+ mice expressed higher levels of LRP1, when compared to the livers from TTR−/− animals (Fig. 7C1). Protein analysis confirmed the effect of TTR at increasing LRP1 and as for the brains, significant differences were observed between TTR+/+ and TTR−/− mice (Fig. 7C2). As for the cell line, SAHep cells analyzed by qRT-PCR (Fig. 7D1) and immunocytochemistry (Fig. 7D2) showed increased LRP1 mRNA and protein levels, respectively, when incubated with TTR.

Altogether, these results indicate that TTR regulates LRP1 levels, suggesting that TTR uses this receptor to promote Aβ clearance.

## Discussion

TTR is a transporter protein mainly synthesized in the liver and in the CP of the brain and secreted into the blood and CSF, respectively. TTR is known to transport several molecules, in particular T4 and retinol through binding to the retinol binding protein (RBP). In the CSF, TTR binds Aβ peptide impeding its deposition in the brain. However, the molecular mechanism underlying this process is not known. Given our earlier evidences that TTR lowers brain and plasma Aβ^11^, we hypothesized that TTR could function as an Aβ carrier that transports the peptide to its receptor at the brain barriers and at the liver.

Since the cerebral capillaries represent about the double of the total apical surface area of the CP^27^, we decided to start by studying the effect of TTR in Aβ transport at the BBB. Using the hCMEC/D3 *in vitro* model of the BBB, we showed that TTR significantly increased Aβ internalization by these cells. Both in the presence and absence of TTR, Aβ internalization levels were high after 15 minutes and no significant increase was measured after 30 minutes. Thus, we assessed efflux by removing media with FAM-Aβ1-42 after a period of incubation to show that TTR was also promoting Aβ efflux from these cells.

To further study the effect of TTR in Aβ transport using the hCMEC/D3 model and given the differential expression of receptors in polarized BBB endothelial cells, we next performed our experiments using transwell cultures. Brain-to-blood transport of Aβ peptide was investigated and we concluded that TTR increased Aβ transport, if added to the brain side but not if added to the blood side. This observation is consistent with a direct TTR/Aβ interaction, as previously demonstrated^28^. To understand if TTR was also being transported while carrying Aβ, we also evaluated TTR ability to cross the endothelial monolayer to show that this protein can cross in the brain-to-blood direction, but does not cross in the opposite direction. To confirm this, we analyzed *in vivo* TTR brain permeability using TTR−/− mice injected with h rTTR either into the brain ventricle or into the tail vein. The presence of TTR was then investigated in brain and blood. The results corroborated the *in vitro* observations since upon IC administration of TTR, the protein was rapidly found in blood; however, after IV injection of TTR the protein was detected neither in CSF nor in the brain extracts. Our findings are also supported by previous work on TTR turnover and degradation^29^; in this work authors reported that rat TTR injected intraventricularly into the CSF of rats was mainly degraded in the liver and kidneys (therefore effluxing from the brain), whereas no specific transfer of plasma TTR to the nervous system or degradation of plasma TTR in the nervous system was observed. It is worthy to note that Makover and colleagues injected purified rat TTR in a system containing the same endogenous rat TTR^29^, and results are similar to the ones we describe now. Therefore, we can conclude that in our system the TTR−/− background did not significantly affected TTR clearance.

The differential brain permeability to TTR indicates the use of a receptor with preferential expression on the basolateral membrane of the endothelial cells forming the BBB, such as LRP1, which in turn is known to internalize Aβ peptide. Whether TTR can cross or not as a complex, namely with Aβ peptide, is not known and needs to be investigated.

TTR gene expression in the brain is usually described as being confined to the CP and meninges, although TTR can be transported to other brain cells. For instance, it is described that in situations of compromised heat-shock response, and as a response to cerebral ischemia, CSF TTR contributes to control neuronal cell death, edema and inflammation^12^. This implies that TTR is transported from CSF to other brain areas, and thus it is also possible that this protein participates in Aβ transport at the BBB. TTR gene expression has been also attributed to neurons and for instance, SH-SY5Y cells transfected with APP695 isoform showed up-regulation of TTR mRNA expression, with concomitant decrease in Aβ levels^16^. Other authors showed that the majority of hippocampal neurons from human AD and all those from APP23 mouse brains contain TTR. In addition, quantitative PCR for TTR mRNA and Western blot analysis showed that primary neurons from APP23 mice transcribe TTR mRNA, and that the cells synthesize and secrete TTR protein^15^. More recently, it has been shown that TTR transcription and protein production can be induced by heat shock factor 1 (HSF1) in hippocampal neurons but not in the liver, both using cell lines and *in vivo* approaches^17^.

Importantly, the BCSFB should also be investigated for TTR-assisted Aβ transport, since this protein is the major protein binding Aβ in CSF. In spite of the low TTR levels in CSF (~2 mg/mL), the choroid plexus is presented as the major site of TTR expression, expressed as a ratio of TTR/mass of tissue, corresponding to a ~30-fold higher than that found in plasma^30^. Interestingly, a recent report describes that in a triple transgenic mouse model of AD only the Aβ1-42 isoform is increased at the epithelial cytosol, and in stroma surrounding choroidal capillaries. Noteworthy, there was increased expression, presumably compensatory, of the choroidal Aβ transporters: LRP1 and RAGE. In addition, authors reported that the expression of TTR was attenuated as compared to non-transgenic mice^31^.

Previous works indicated that the genetic reduction of TTR in an AD mouse model results in increased Aβ brain levels^9^,^10^; another work using 7 month old female mice also showed increased Aβ1-42 plasma levels in AD/TTR+/− mice as compared to age-and gender-matched AD/TTR+/+ animals. In the present work, we extended our study and evaluated both plasma Aβ1-42 and Aβ1-40 isoforms in 3 months old AD/TTR+/+, AD/TTR/+/− and AD/TTR−/− animals, showing that TTR correlates negatively with both isoforms of Aβ. Further, these findings support the idea that plasma may also reflect disease disturbances in AD.

Thus, the following level of our study focused on the effect of TTR in Aβ peptide uptake by the liver. After showing that h rTTR produces a concentration-dependent increase in Aβ internalization by SAHep cells, we worked with primary hepatocytes derived from mice with different TTR backgrounds showing again higher levels of internalization in the presence of TTR.

Interestingly, previous work has shown that TTR is internalized by the liver using a RAP-sensitive receptor^20^, such as LRP1. Multiple factors influence the function of LRP1-mediated Aβ clearance, such as its expression, shedding, structural modifications and transcriptional regulation by other genes^32^. Recent studies have clarified how Aβ clearance mechanisms in the CNS are indirectly altered by vascular and metabolism-related genes via the sterol regulatory element binding protein (SREBP2)^33^. In addition, AD risk genes such as phosphatidylinositol binding clathrin assembly protein (PICALM)^34^ and apoE isoforms can differentially regulate Aβ clearance from the brain through LRP1^35^.

Consequently, given the importance of this receptor in Aβ clearance both from the brain and at the liver, we evaluated the levels of gene and protein expression in different models. Both LRP1 transcript and protein levels were increased in TTR+/+ brains as compared to TTR−/−. To further confirm the importance of TTR in regulating the levels of LRP1 specifically at the BBB, and contributing to explain the importance of TTR in Aβ clearance, we measured LRP1 in hCMEC/D3 cells with and without incubation with TTR. We observed that the presence of TTR clearly increased the receptor expression, producing significant differences. A similar study was then undertaken for liver and SAHep cells, which again showed regulation of LRP1 expression by TTR. Whether liver TTR regulates liver LRP1 and CSF TTR regulates brain LRP1 is not known and further studies, namely differential silencing of the TTR gene (liver or CP), should be performed.

In a recent study, TTR has been described to regulate insulin-like growth factor receptor I (IGF-IR) expression in mouse hippocampus (but not in choroid plexus) and this effect is due to TTR mainly synthesized by the choroid plexus (and secreted into the CSF) and not by peripheral TTR^36^. Once more, the possibility for local TTR production has been advanced by some authors^16^,^17^, as already mentioned. Finally, it is also known that LRP1 and IGF-IR interact^37^,^38^ in a way that the extracellular ligand-binding domain of LRP1 is not involved thus remaining free to bind its ligands. A common link is now established as TTR can regulate the expression of both receptors, albeit in different areas of the brain, opening the possibility for TTR being involved in other processes in the CNS. Moreover, using mice with deleted APP and APLP2, APP has been shown to down-regulate expression of LRP1^39^ via epigenetic events mediated through its intracellular domain (AICD) and to up-regulate TTR, as previously described^16^. Though it is not known if LRP1 and TTR regulation are part of the same AICD-pathway since TTR levels were not evaluated in the APP and APLP2-deleted mice.

In summary, we show that neuroprotective effects of TTR previously observed in the context of AD are consistent with its role in Aβ clearance at the BBB and liver, and that TTR regulates LRP1 expression, suggesting that TTR is also transported by this receptor. In the future, the TTR-LRP1 cascade should be further investigated for therapeutic targeting.

## Additional Information

**How to cite this article**: Alemi, M. *et al*. Transthyretin participates in beta-amyloid transport from the brain to the liver- involvement of the low-density lipoprotein receptor-related protein 1? *Sci. Rep*. **6**, 20164; doi: 10.1038/srep20164 (2016).

## Figures and Tables

**Table 1:** TTR concentrations as measured by ELISA after IC or IV injection in TTR−/− mice.

	TTR concentration (ng/mL)				
	Brain (after 60 min)	CSF (after 60 min)	Plasma		
Tested Mice			20 min	40 min	60 min
Control mouse (non-injected/showing background levels)	28,7	88,4	—	—	2,1
IC injection of TTR					
Mouse 1	488	—	729,5	983,1	1005,7
Mouse 2	672,2	—	754,8	1480,0	817,3
IV injection of TTR					
Mouse 3	23,9	19,2	707,1	1011,4	1008,5
Mouse 4	71,5	12,1	855,3	1079,7	615,2
